# Substantial reduction of inappropriate tablet splitting with computerised decision support: a prospective intervention study assessing potential benefit and harm

**DOI:** 10.1186/1472-6947-9-30

**Published:** 2009-06-12

**Authors:** Renate Quinzler, Simon PW Schmitt, Maria Pritsch, Jens Kaltschmidt, Walter E Haefeli

**Affiliations:** 1 Department of Internal Medicine VI, Clinical Pharmacology and Pharmacoepidemiology, University of Heidelberg, 69120 Heidelberg, Germany; 2 Institute of Medical Biometry and Informatics, University of Heidelberg, 69120 Heidelberg, Germany

## Abstract

**Background:**

Currently ambulatory patients break one in four tablets before ingestion. Roughly 10% of them are not suitable for splitting because they lack score lines or because enteric or modified release coating is destroyed impairing safety and effectiveness of the medication. We assessed impact and safety of computerised decision support on the inappropriate prescription of split tablets.

**Methods:**

We performed a prospective intervention study in a 1680-bed university hospital. Over a 15-week period we evaluated all electronically composed medication regimens and determined the fraction of tablets and capsules that demanded inappropriate splitting. In a subsequent intervention phase of 15 weeks duration for 10553 oral drugs divisibility characteristics were indicated in the system. In addition, an alert was generated and displayed during the prescription process whenever the entered dosage regimen demanded inappropriate splitting (splitting of capsules, unscored tablets, or scored tablets unsuitable for the intended fragmentation).

**Results:**

During the baseline period 12.5% of all drugs required splitting and 2.7% of all drugs (257/9545) required inappropriate splitting. During the intervention period the frequency of inappropriate splitting was significantly reduced (1.4% of all drugs (146/10486); p = 0.0008). In response to half of the alerts (69/136) physicians adjusted the medication regimen. In the other half (67/136) no corrections were made although a switch to more suitable drugs (scored tablets, tablets with lower strength, liquid formulation) was possible in 82% (55/67).

**Conclusion:**

This study revealed that computerised decision support can immediately reduce the frequency of inappropriate splitting without introducing new safety hazards.

## Background

Tablet splitting is an indispensable method for dose individualisation and a common strategy to save medication costs [1-5]. In Germany ambulatory patients split about one fourth of their tablets before ingestion [6]. However, roughly 10% of split tablets are not suitable for splitting because they lack score lines or because enteric or modified release coating precludes safe breaking [6,7]. Such medication errors are mainly initiated by physicians during the prescription process [8] and similar to crushing of tablets might reduce the effectiveness of drug treatment or promote adverse events if enteric or modified coating is destroyed [9-11]. Moreover, tablet splitting may require intensified patient counselling [12]. An essential fraction of these medication errors is considered preventable because more suitable drugs (i.e. scored tablets or tablets with lower strengths) are available. However, in the legal prescribing information, the Summary of Product Characteristics (SPC), only limited information on divisibility is available [6,7] and therefore inappropriate tablet splitting is difficult to prevent.

In 2003 we developed and implemented an electronic prescription system (AiD*Klinik*) at the University Hospital of Heidelberg. It provides up-to-date information on all drugs marketed in Germany and is equipped with a prescription platform that allows composing patient medication regimens for inclusion in discharge letters or print-out on prescription forms. Because in this application physicians may specify individual dosing regimens this platform provides an excellent opportunity to intercept excessive doses, dangerous drug interactions, and also inappropriate tablet splitting during the prescription process. We therefore equipped this system with a large database containing splitting information and assessed the impact of computerised decision support to prevent inappropriate prescription of split tablets and capsules. We also evaluated the safety of the electronic intervention because the implementation of new health technology has been associated with the potential risk of introducing new and even fatal errors [13-17].

## Methods

After approval by the Ethics Committee of the Medical Faculty of the University of Heidelberg we performed a prospective intervention study in a 1680-bed university hospital providing primary and tertiary care to an urban population. Over a period of 15 weeks we collected all medication regimens composed with the electronic prescription system for ambulatory patients or patients at discharge. In the subsequent intervention phase of 15 weeks duration, the electronic prescription system provided structured information on divisibility of solid oral dosage forms in two ways. For each drug with splitting information (n = 10553 tablets and capsules) icons were displayed in the drug information system. These icons indicated whether (i) the drug was a capsule or a tablet, whether (ii) score lines were present, and (iii) whether dividing into two, three, or four fragments was possible. In addition, an alert was generated whenever a drug including corresponding dosage regimen was entered in the prescription platform demanding inappropriate splitting (splitting of capsules, of unscored tablets, or of tablets with score lines unsuitable for the intended fragmentation). Subsequently, physicians could modify the dosage regimen, select another drug, or insist on the original (unsuitable) dosage regimen.

In each study phase we evaluated the fraction of inappropriately split solid oral drug formulations. We assessed the appropriateness of splitting using a modified database (Pharmindex, MMI der Wissensverlag, Neu-Isenburg, Germany) containing information on the splitting properties of 10553 brands marketed in Germany (tablets and capsules). To avoid bias, we included medication regimens in the analysis only from clinics and wards that contributed electronically written medication regimens in both study phases. Furthermore, we included only drugs with an unequivocal dosage regimen and splitting information in our database if they were marketed during the whole study phase.

Additionally, to assess the impact of alerting we logged during the prescription process all drugs and corresponding dosage regimens prompting an alert and compared them with the final medication regimens of the individual patient. We analysed how often in response to an alert (i) another brand or drug with different strength was selected, (ii) the dosage regimen was adjusted, (iii) no changes were made regardless of the alert, or (iv) the drug was removed from the prescription platform without substitution. We also assessed whether the alert may have unintended negative effects on prescribing quality. Therefore, for every change in a medication regimen subsequent to an alert we analysed the final medication with respect to three different scenarios: (i) the dosage form of the selected alternative was inappropriate (e.g. the selected alternative was neither appropriate for splitting). (ii) After adjustment the dosage regimen was no longer in accordance with the dosage recommendations of the corresponding SPC with respect to dosage interval and/or maximum recommended dose. Finally, (iii) the adjusted dosage regimen demanded the intake of an evitable large number of tablets (e.g. two 5 mg tablets instead of one 10 mg tablet) and/or prescription of an evitable large number of drug products with different strengths (e.g. five 1 mg tablets instead of a split 10 mg tablet).

On the basis of a pilot analysis of previous medication log files it was estimated that each day about 90 drugs eligible for analysis are entered, about 10% of all tablets and capsules are split, 15% of the split drugs are not suitable for splitting, and the intervention will lead to a 30% reduction of the number of inappropriately split drugs. Therefore, a duration of 15 weeks of each study period was planned to achieve a power of 0.8 to detect this difference between p1 = 0.015 and p2 = 0.0105 of all eligible tablets applying a two-sided χ^2^-test with a significance level of α = 0.05 (sample size: 9757 per period). Since no data were available on the correlation structure between the prescriptions within one physician or within one ward this dependency could not be inluded in the sample size estimation. In the analysis, however, the wards were considered as clusters and the dependency was accounted for in a logistic regression model applying the method of generalised estimating equations with assumed exchangeable correlation structure [18,19]. Period was the only included influencing factor in the model and tested two-sided with a significance level of α = 0.05. The analysis was carried out using the Statistical Analysis System, Version 9.1 for Windows (SAS Institute Inc., Cary, NC, USA), for sample size calculation nQuery Advisor 7.0 was used.

## Results

Electronic medication regimens of 54 wards and clinics of the University Hospital of Heidelberg fulfilled the inclusion criteria and were collected between August 2006 and March 2007. These wards contributed 29517 electronically prescribed drugs. Not eligible for analysis and therefore excluded were formulations other than capsules or tablets (n = 6304; 21.4%), drugs without information on divisibility in our database (n = 1501; 5.1%), drugs that were not marketed during the whole study phase (n = 15; 0.1%), and drugs with equivocal dosage regimens (n = 1666; 5.6%). Finally, 20031 tablets and capsules were eligible for analysis (baseline period: 9545 drugs; intervention period: 10486 drugs). The most frequently prescribed drug groups were (1^st ^ATC level; percentage of drugs prescribed in the baseline period and intervention period, respectively): drugs used in the cardiovascular system (C; 33.2%; 30.4%), alimentary tract and metabolism (A; 23.7%; 22.7%), nervous system (N;12.9%; 16.0%), and antiinfectives for systemic use (J; 8.3%; 8.8%).

### Benefit of the intervention

During the baseline period, in which no splitting information was displayed in the electronic prescription system, 12.5% of all drugs were prescribed in split form. In 2.7% of all drugs splitting was inappropriate for the following reasons: split preparations were unscored tablets (71.2%), or capsules (15.6%), or scored tablets did not allow the desired partitionment (13.2%; Table 1).

**Table 1:** Frequency of (inappropriate) splitting of tablets and capsules before and during computerised decision support.

Prescribed dosage form	Baseline period		Intervention period	
	**n**	**(%)**	**n**	**(%)**

Split scored tablets/all scored tablets	966/5126	(18.8)	976/5876	(16.6)

Inappropriately split scored tablets/all scored tablets	34/5126	(0.7)	33/5876	(0.6)

(Inappropriately) split unscored tablets/all unscored tablets	183/3386	(5.4)	76/3496	(2.2)

(Inappropriately) split capsules/all capsules	40/1033	(3.9)	37/1114	(3.3)

Total number of split drugs*/all drugs*	1189/9545	(12.5)	1089/10486	(10.4)

Total number of inappropriately split drugs*/all drugs*	257/9545	(2.7)	146/10486	(1.4)

*capsules and tablets

During the intervention period with an immediate feedback alert that interrupted the prescription process 10.4% of all drugs required splitting and 1.4% of all drugs were inappropriate for splitting as prescribed (Table 1). Hence, the intervention nearly halved (odds ratio: 0.51, 95%-CI: 0.35–0.76, χ^2 ^= 11.23, p = 0.0008) the proportion of inappropriately split drugs mainly by intercepting undue fragmentation of unscored tablets.

### Impact of alerting

During the intervention period 136 alerts, which immediately informed prescribers about dosage regimens demanding inappropriate splitting, were logged and analysed. During the prescription process in response to half of the alerts (69 of 136) the prescribers made adjustments (selection of another brand, adjustment of dosage regimen) and in the overwhelming majority (87%, 60 of 69) the adjustments clearly improved the medication regimen (Table 2). In the other half of alerts (67 of 136) no corrections were made (n = 58), the dosage regimen prompting the alert was removed (n = 7), or the drug was removed from the dosage regimen (n = 2) although a switch to more suitable drugs (scored tablets, tablets with lower strength, liquid formulation) would have been possible in 82% (55 of 67) of the cases.

**Table 2:** Evaluation of measures taken during the prescription process in response to an alert.

Adjustment of medication regimen in response to an alert	Total number of adjustments n (%)	Suboptimal adjustments	Number of suboptimal adjustments n
Switch to another product (same or lower strength)	26 (37.7)	Prescription still demands splitting of unscored brand	4

Switch to ≥ 2 brands with different strengths	16 (23.2)	Suboptimal combination of strengths (preventable large number of tablets has to be taken)	3

Selection of a liquid formulation	1 (1.4)	Selection of parenteral solution instead of oral solution	1

Adjustment of dosage regimen (dosage interval and/or daily dose)	23 (33.3)	maintenance dose was increased and exceeds the maximum recommended dose in the SPC	1

Selection of another brand and adjustment of dosage regimen	3 (4.3)	none	0

Total	69 (100)		9

## Discussion

This study revealed that an important fraction of medication regimens prescribed at discharge demands inappropriate splitting of tablets and capsules and therefore carries the risk of careless destruction of galenic forms potentially translating into adverse events and treatment failure [9-11]. This intervention demonstrated that computerised decision support can immediately improve prescriber performance and reduce the frequency of inappropriate tablet splitting. Over half of the alerts prompted an adjustment of the medication regimens indicating that the user took the warnings seriously. However, not all adjustments clearly improved the medication regimens and almost as frequently the physicians insisted on the primary (inappropriate) dosage regimen although in most cases a switch to more appropriate drugs would have been possible.

Although generally expected to be beneficial and innocuous, computerised decision support systems have also been shown to introduce new errors and sometimes even to cause harm when used in a hospital [13-17]. It is therefore increasingly important to carefully study their impact under real life conditions before dissemination [20]. In this study we also aimed at evaluating whether the intervention may have triggered new errors and therefore scrutinised all medication regimens that were adjusted in response to the alert. While the overwhelming majority of changes led to an improvement of the medication regimens, in 13% the adjustments were not satisfactory because (i) the subsequently prescribed drug was not suitable for splitting either, (ii) the dosage regimen (still) infringed upon recommendations in the drug label, (iii) an unnecessarily large number of tablets had been prescribed, or (iv) an unsuitable dosage form was selected. These findings therefore suggest that the tool substantially improved prescription quality without introducing new risks. The findings also indicate that physicians need additional support in the process of tablet splitting. In this intervention the alert simply warned when inappropriately split tablets were prescribed but the system did not suggest more suitable alternatives. Whether switching can be further encouraged and improved by suggesting more appropriate drugs to the user concurrently with the alert (e.g. scored tablets, tablets with the intended strength, or liquid formulations) will have to be studied.

A limitation of this study may be that in Germany and elsewhere general practitioners frequently change patients' medication regimens after discharge [21] and therefore we cannot determine whether these medication regimens were indeed prescribed to the patients as suggested in the discharge letter. However, our earlier study in ambulatory German patients [6] revealed that inappropriate splitting was in a similar order of magnitude suggesting that support is needed in both sectors of the health care system.

## Conclusion

In conclusion, an important fraction of medications prescribed at hospital discharge demands inappropriate splitting of tablets and capsules. Computerised decision support eliminates half of these errors without introducing new risks.

## Competing interests

RQ: None declared

SPWS: None declared

MP: None declared

JK is CEO of Dosing GmbH Heidelberg.

WEH reports having received lecture fees from Altana, Astellas, Berlin-Chemie, Boehringer-Ingelheim, Novartis, Sankyo and consulting fees from Bayer and Roche.

## Authors' contributions

RQ: conceived and designed the study, analysed and interpreted the data, and drafted the manuscript.

SPWS: participated in the conception of the study, developed and implemented the decision support system, and critically revised the manuscript

MP: analysed the data and revised the manuscript critically

JK: developed AiD*Klinik*, supervised the implementation of the decision support system, and critically reviewed the manuscript

WEH: developed AiD*Klinik*, conceived and designed the study, participated in the analysis and interpretation of the data, and drafted the manuscript

All authors read and approved the final manuscript

## Pre-publication history

The pre-publication history for this paper can be accessed here:

http://www.biomedcentral.com/1472-6947/9/30/prepub
